# 3D Extracellular Matrix Regulates the Activity of T Cells and Cancer Associated Fibroblasts in Breast Cancer

**DOI:** 10.3389/fonc.2021.764204

**Published:** 2021-12-09

**Authors:** Huan Gao, Qi Tian, Lizhe Zhu, Jinteng Feng, Yan Zhou, Jin Yang

**Affiliations:** ^1^ Department of Medical Oncology, The First Affiliated Hospital of Xi’an Jiaotong University, Xi’an, China; ^2^ Department of Breast Surgery, The First Affiliated Hospital of Xi’an Jiaotong University, Xi’an, China

**Keywords:** extracellular matrix (ECM), cancer associated fibroblast (CAF), T cell activation, tumor microenvironment, breast cancer

## Abstract

**Background:**

Breast cancer progression has been gradually recognized as a bidirectional interaction between cancer cells and tumor microenvironment including stroma cells, immune cells, and the dynamically altered ECM. However, there still lacks direct experimental evidences about how ECM properties modulate the activities of stroma and immune cells.

**Method:**

The transcriptomic data and corresponding clinical information of breast cancer pawere obtained from TCGA. Patients were divided into ECM-high, ECM-median and ECM-low groups based on ssGSEA scores of C-ECM genes. The prognostic value of ECM was confirmed by univariate/multivariate Cox regression and survival analyses. GO and KEGG analyses were performed between ECM-high and -low groups. Then associations between ECM characteristics and clinical stages were verified by Masson’s trichrome and Sirius red/Fast Green staining of clinical breast cancer tissues. To evaluate the effects of ECM on CAF induction and T cell activation, the MRC-5, NIH/3T-3, primary T cells and Jurkat T cells were encapsulated in 3D collagen with different densities and organizations, and the expression levels of CAF biomarkers and secretion levels of IL-2 were assessed.

**Results:**

ECM scores showed broad variation across paracancerous and cancer samples as well as breast cancer molecular subtypes, and patients with different ECM groups showed distinct prognosis. Immunological activity and ECM associated biology processes were identified by GO and KEGG analyses across ECM-high and -low groups. According to MCP-counter algorithm, the infiltration of T cells was significantly lower in the ECM-high group, while CAF abundance was significantly higher. It is furtherly confirmed by clinical samples that collagen density and organization were associate with breast cancer progression. Finally, *in vitro* 3D-cultured fibroblasts and T cells validated that the density and organization of collagen showed significant effects on CAF induction and T cell activation.

**Conclusion:**

Our study revealed a new mechanism of T cell immunosuppression and CAF induction, which could be of central importance for the breast cancer invasion and may constitute novel therapeutic targets to improve breast cancer outcomes.

## Introduction

Breast cancer remains a worldwide health burden that ranks as the leading malignancy (1). Despite the frequent occurrence, the mechanisms of breast cancer are far from being completely understood. For the past several decades, studies of breast cancer focused on tumor cell biology, wherein the hallmarks of cancer emphasized the tumorigenesis process (2). However, tumor metastasis continues to be the main difficulty of breast cancer treatment. With the gradual advancement of exploring, the process of breast cancer development had gradually been recognized as a bidirectional interaction between cancer cells and certain cell types within the tumor microenvironment including stroma cells, immune cells, and the extracellular matrix (ECM) (3). Therefore, tumor microenvironment is generally considered as a coconspirator in tumor initiation and progression.

During breast cancer progression, cells in the microenvironment, especially stroma cells and immune cells, dynamically remodel the ECM network and result in density, stiffness or organization changes (4, 5). More and more researches provide evidences that the remodeled ECM can regulate the bioprocess of breast cancer cells thus promote invasion and metastasis (6, 7). For example, increasing ECM stiffness induces malignant phenotypes of mammary epithelium cells through Integrin-Rho-YAP/TAZ dependent pathway (6, 8). Besides, quantifying collagen alignment has also been confirmed as a novel prognostic marker for the breast cancer survival (9). However, it remains quite speculative whether the ECM characteristics also modulate the activities of other cells to influence tumor microenvironment and thereby support breast cancer invasion (10).

Tumor infiltration lymphocytes, and in particular CD8+ cytotoxic T cells, is known to predict good prognosis and immunotherapy responses in breast cancer (11). Meanwhile, cancer associated fibroblasts (CAFs), the most prominent stromal components and major producer of ECM (12), have been shown to contribute to drug resistance and breast cancer immunity suppression (13). Daniel D. De Carvalho el al. identified a distinct pan-cancer transcriptional pattern of ECM genes based on combined database analysis, termed as C-ECM genes and found that the ECM dysregulation influence CAF induction *via* regulating the activation of TGF-β signaling pathway (14). Furthermore, Daniel H. Madsen et al. employed 3D culture assays and observed collagen-density can directly impact the activity of T cells (15). Importantly, the above studies revealed that the ECM also plays a regulatory role in the activity of T cells and CAFs. However, it was not addressed in these studies what kinds of specific ECM characteristics showed prognostic values and if the collagen organization also influenced the activity of the T cells and CAFs. Besides, there still lacks of combined experimental evidences.

Obviously, new work should focus on identifying specific ECM features related to breast cancer invasion and prognosis in clinical samples, and evaluating how these specific ECM characteristics regulate the activities of T cells and CAFs spatiotemporally. In the present study, we investigate the specific ECM characteristics associated with breast cancer invasion and prognosis based on combined analysis of clinical samples and TCGA database. As collagen density and organization showed prognostic value according to the combined analysis, we then investigate how 3D collagen density and organization affect the activities of T cells and fibroblasts treated with the supernatant samples of breast cancer MB-231 cells. We observed that increased collagen density and alignment promote the CAF induction and suppressed T cell activation. Importantly, our finding may inspire a new cancer treatment strategy by targeting ECM density and organization, which may help to open a new window for studies on breast cancer invasion.

## Method

### Collection and Grouping of Breast Cancer Data

The fragments per kilobase of per million (FPKM) of breast cancer transcriptome were downloaded from TCGA database (https://portal.gdc.cancer.gov). A reference gene set of cancer extracellular matrix associated (C-ECM) genes including 30 significant upregulated genes and 28 significant downregulated genes in pan-cancer tissues was derived from Ankur Chakravarthy et al (14). ECM-up and –down scores were carried out by using ssGSEA method of R software Gene Set Variation Analysis (GSVA) package. According to the clinical data of breast cancer samples in the TCGA, the associations between ECM scores (ECM-up and –down scores) and breast cancer molecular subtypes were evaluated. Then the ECM up and -down scores are classified according to the median. We take the intersection of the samples that are greater than the median in the up-regulation score and those are less than the median in the down-regulation score, and define it as the ECM-high group. At the same time, we define the ECM-low group by taking the intersection of the samples that are less than the median in the up-regulated score and those that are greater than the median in the down-regulated score, while those samples, in which ECM-up scores higher than the median coexist with ECM-down scores lower than the median or ECM-up scores lower than the median coexist with ECM-down scores higher than the median, were defined as ECM-median group.

### Verification of the Effectiveness of ECM Grouping

We compared the overall survival (OS) and disease free survival (DFS) of breast cancer patients in ECM-high, -median and –low subtype by R software “survival” package. The log-rank test was performed to test the survival differences. Kaplan-Meier curves were used to visualize the OS and DFS of different ECM subtypes. Univariate and multivariate Cox regression analysis regarding on OS was performed to determine the hazard ratio (HR) of each ECM subtype by using the “survival” package of the R software. The sensitivity and specificity of the ROC curve were conducted to evaluate the prognostic value of the ECM subtypes.

### Pathway Analysis of ECM Phenotype

The “edgeR” package calculation in R software was used to perform a differential analysis of the mRNAs of ECM-high and –low groups according to the cutoff screening of |log2FC| > 1 and *p* <0.05. Gene ontology (GO) terms including biological processes (BPs), cellular components (CCs), and molecular functions (MFs) were analyzed by R “clusterProfiler” package to perform biological functions of differentially expressed genes (DEGs) between ECM-high and –low groups. To explore the possible signaling pathways DEGs enriched, KEGG enrichment analysis was carried out utilizing the “clusterProfiler” package of R software with a statistical threshold of *p* < 0.05.

### Micro-Environmental Analyses of ECM Dysfunction

The relationship between ECM dysfunction and microenvironment status was analyzed on TIMER2.0 (http://timer.cistrome.org/). This freely available web provides more robust estimation of immune infiltration levels for TCGA or user-provided tumor profiles using six state-of-the-art algorithms. Microenvironment Cell Populations-counter (MCP-counter) algorithm was selected for quantification of the absolute abundance of eight immune and two stromal cell populations in each group (16).

### ECM Characterization of Clinical Patients

In order to clarify the characteristics of the ECM corresponding to each ECM phenotype, we performed Masson’s trichrome staining (Solarbio, China) and Sirius red/Fast Green staining (Solarbio, China) to assess the ECM characteristics of breast cancer patients of different nodal status and pathological stages according to the manufacturer’s instructions. The density of collagen was estimated using red-stained collagen area of Sirius red/Fast green staining and blue-stained collagen area of Masson’s trichrome using Image J in ten randomly selected microscopic fields per specimen. The degree of collagen fiber alignment was quantified using an image J plugin Orientation J.

### 3D Culture in Different Collagen Concentration Gels

All the animal studies conformed to the guidelines set by the Institutional Animal Care Committee of Xi’an Jiaotong University. Collagen fibers were harvested from rat tail tendons and labeled with fluorescein isothiocyanate (FITC) (Yeasen, China) as described previously (17, 18). The human breast cancer cell line MDA-MB-231, the mouse embryo fibroblast cell line NIH/3T-3 and human embryonic lung fibroblast cell line MRC-5 were obtained from American Type Culture Collection (ATCC, Manassas, VA) and cultured in DMEM supplemented with fetal bovine serum (FBS; Gibco, Grand Island, NY, USA), penicillin, and streptomycin (Sigma, St. Louis, MO, USA). Primary human T cells were obtained as a gift from Dr Jinteng Feng and Jurkat T cells were purchased from the Cell Bank of the Chinese Academy of Sciences (Shanghai, China). The culture medium of primary T and Jurkat T cells were RPMI 1640 (Hyclone, Logan, UT, USA) supplemented with FBS, penicillin, and streptomycin. The supernatant of MB-231 cells was added to fibroblasts and T cell culture medium at a 1:1 ratio for 12 hours during 3D culturing. Primary T and Jurkat T cells were stimulated by Dynabeads™ Human T-activator CD3/CD28 (ThermoFisher, 11161D) for 24h before co-cultured with the supernatant of breast cancer cells. T cells and fibroblasts were capsulated in low and high density gels contained 1mg/ml and 4 mg/ml type I collagen at a concentration of 6×10^5^ cells/gels respectively. Both cell types were used between passages 2–20 and all cells were maintained at 37°C in a humidified 5% CO2 atmosphere.

### 3D Culture in Magnetic Force-Induced Aligned Collagen Gels

Cells capsulated in collagen gels were injected into a dog bone-like mold. 10 iron oxide microspheres with diameter of ~200 μm were placed into both edges of collagen suspension. To obtain aligned collagen fibers, two NdFeB magnets (6 mm × 1.5 mm) were used to apply 10% uniaxial strain to the gels for 24h.

### Immunofluorescence Staining and Confocal Microscopy

Cells were encapsulated in collagen gels with different collagen concentration or fiber organization. Then rinsed, fixed in 4% formaldehyde and permeabilized for 10 min with 0.5% Tritonx-100, and incubated in 1% BSA for 1 hour followed by incubation with 1:500 dilution of anti-alpha smooth muscle actin antibody (ab7817, Abcam, 4°C, overnight) and fibronectin monoclonal antibody (14-9869-82, invitrogen, 4°C, overnight), then incubated with secondary antibody (Alexa Fluor^®^ 647, Invitrogen, room temperature, 2hs), and 4′,6-Diamidino-2-phenylindole (DAPI, Invitrogen) was used to stain cell nuclei. Confocal fluorescence microscopy was performed using Olympus FV300. The fluorophores were excited by 405, 488 and 647 nm laser lines.

### Enzyme Linked Immunosorbent Assay (ELISA) to Measure IL-2 Concentration

Supernatant samples were collected 48h after encapsulation of cells in collagen gels and assayed for IL-2 levels using a commercial IL-2 ELISA Reagent Kit (Fisher scientific) following the product protocol. Measure absorbance on an ELISA plate reader set at 450nm with microplate reader (Thermo Fisher) and use the provided Standard Diluent to prepare standard curve serial dilutions.

### Statistical Analysis

All data were analyzed by R version 3.6.3 or Graphpad Prism 8, and all cell culture experiments were performed at least three times independently. These results were presented as mean ± standard deviation (SD). Student’s two-sided t-test was used to compare the differences between two groups. Differences in survival between different risk groups were compared by Kaplan-Meier curves followed by log-rank test. P < 0.05 was considered as statistically significant.

## Results

### C-ECM Dysregulation Is Associated With the Prognosis of Breast Cancer

**Figure 1:**
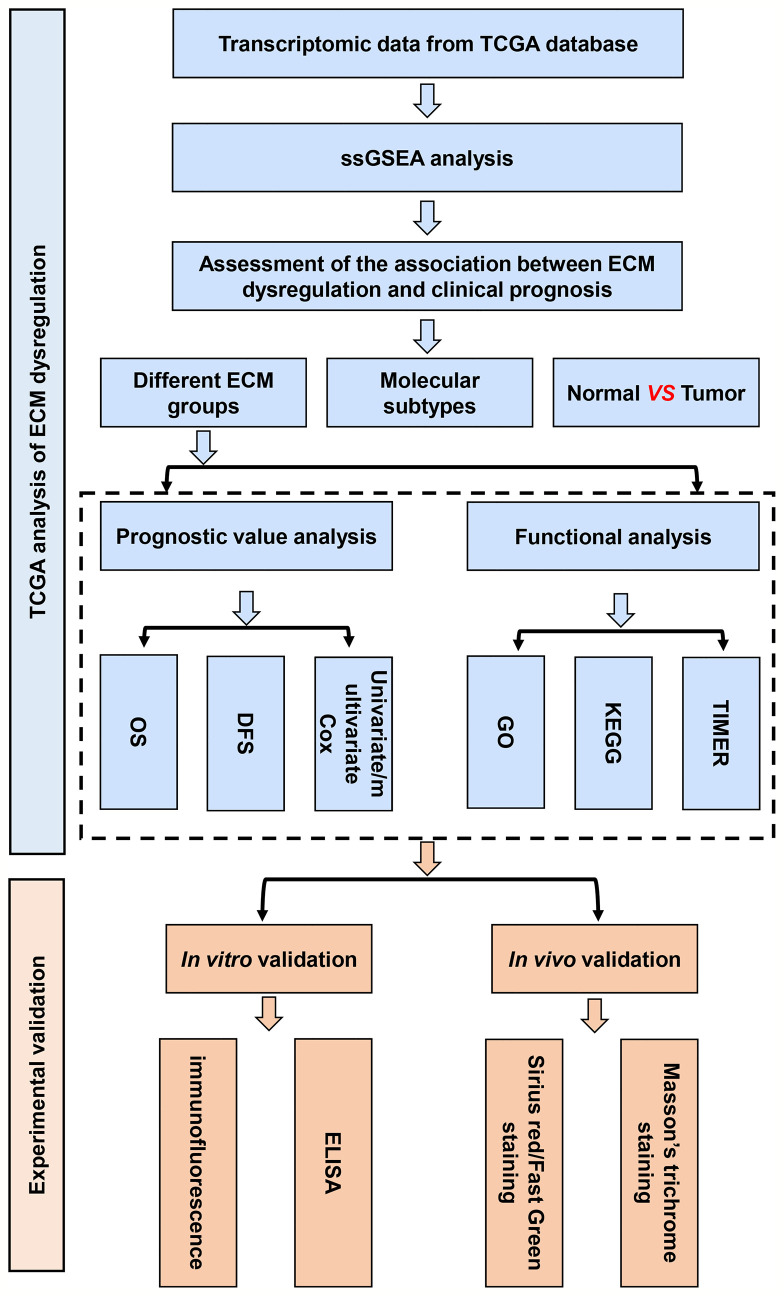
The flow chart of construction and validation of data collection and analysis.

**Figure 2:**
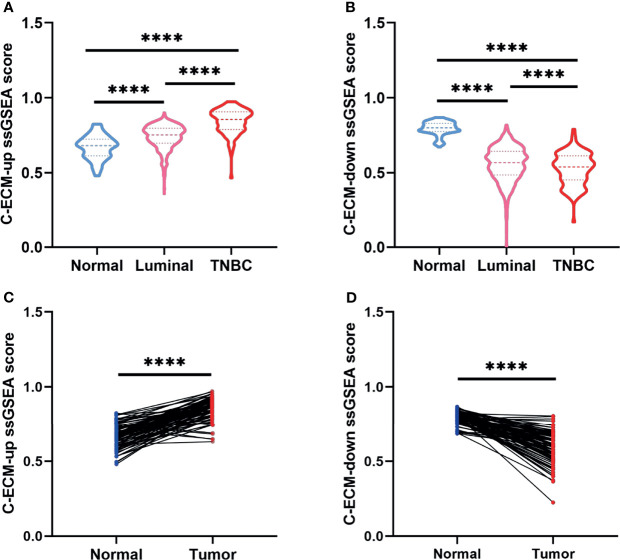
C-ECM dysregulation is associated with the prognosis of breast cancer in TCGA database. **(A)** C-ECM-up ssGSEA scores of normal, luminal, and triple negative (TNBC) breast cancer tissues in TCGA database. **(B)** C-ECM-down ssGSEA scores of normal, luminal, and TNBC breast cancer tissues in TCGA database. **(C)** C-ECM-up scores of paired paracancerous and cancer tissues of breast cancer patients in TCGA database. **(D)** C-ECM-down scores of paired paracancerous and cancer tissues of breast cancer patients in TCGA database. ****P < 0.0001.

Initially, to study whether ECM characteristics showed significant effects in invasion and prognosis of breast cancer, a total of 1109 breast cancer samples and 113 paracancerous samples from TCGA were obtained. The entire flow chart of the research is presented in **Figure 1**, and the clinical information of patients in TCGA database was list in **Table 1**. Upon summarization using single sample Gene Set Enrichment Analysis (ssGSEA) scores to evaluation ECM characteristics, C-ECM-up and -down scores show broad variation across breast cancer molecular subtypes (**Figures 2A, B**). Also, we found that triple negative breast cancer (TNBC) which is considered as the most aggressive molecular subtype showed highest ECM-up scores and lowest ECM-down scores (**Figures 2A, B**). Specifically, compared with the paracancerous samples, cancer samples had higher ECM-up scores and lower ECM-down scores in paired and unpaired breast cancer tissues (**Figures 2A**–**D**).

**Table 1:** Clinical information of breast cancer patients in TCGA database and validation group.

Characteristics	TCGA database	Validation group
Age		
≤60	612	21
>60	369	9
T stage		
T1-T2	924	19
T3-T4	57	11
N stage		
N0	517	12
N1-N3	464	18
Pathological grade		
I-II	NA	14
III	NA	16
ER status		
negative	240	9
positive	741	21
PR status		
negative	346	7
positive	635	23
HER2 status		
negative	335	6
positive	646	24
Molecular subtype		
Luminal	834	23
Triple-negative	113	6
HER2-enriched	34	1

**Figure 3:**
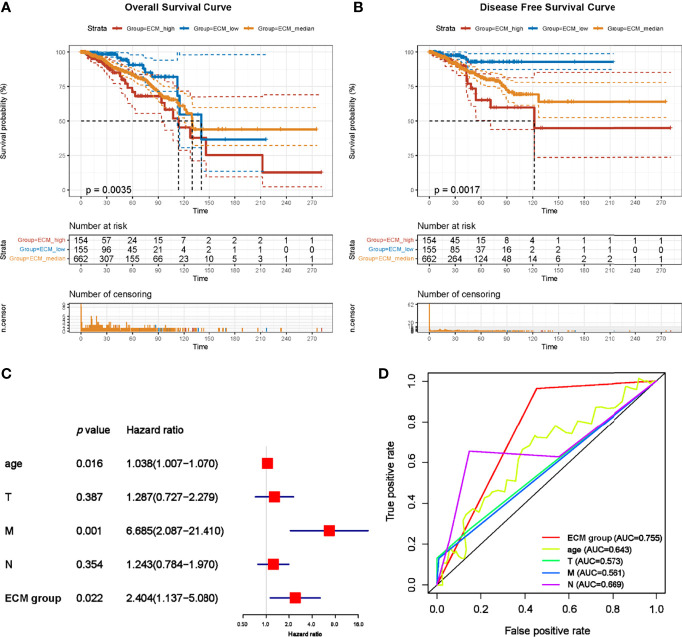
C-ECM dysregulation is associated with the OS and DFS of breast cancer in TCGA database. **(A)** The Kaplan-Meier curves for the OS of patients in TCGA database which were divided into ECM-high, ECM-median and ECM-low group. **(B)** The Kaplan-Meier curves for the DFS of patients in TCGA cohort which were divided into ECM-high, ECM-median and ECM-low group. **(C)** Forest plots of Multivariate Cox regression analysis regarding OS in the TCGA database. **(D)** The AUC of time-dependent multivariate ROC curves was used to verify the prognostic performance of the ECM group in TCGA database.

Subsequently, to estimate whether ECM characteristics contribute to the prognosis of breast cancer, breast cancer patients were divided into 154 ECM-high, 662 ECM-median and 155 ECM-low patients according to ECM-up and –down scores. Kaplan-Meier curves showed that the samples in the ECM-high group exhibited worse overall survival (OS) and disease free survival (DFS) than those in the ECM-median and -low groups, while ECM-low group exhibited the best OS and DFS, indicating the association between ECM dysregulation with breast cancer prognosis (**Figures 3A, B**). Beside, to further explore the prognostic value of the ECM score in each molecular subtypes of breast cancer, we compared the OS and DFS between different ECM subgroups for different molecular subtypes separately. The results of DFS and OS in luminal breast cancer patients were consistent with the general samples (**Figures S1A, B**), however, in TNBC subtype, the ECM-medium group showed the best OS and DFS trends among the three groups with no statistically significant *p*-values (**Figures S1C, D**). Considering the small number of patients in the TNBC subtype, we reclassified these patients into ECM-high and -low groups, then the OS and DFS results began to show a similar trend with ECM-high subgroup showed the worse OS and DFS, while the *p*-values still not statistically significant (**Figures S1E, F**).

We also performed univariate (**Figure S2**) and multivariate Cox regression analyses regarding OS based on ECM group with TNM stage and age of onset to examine the prognostic impact of this dysregulation. ECM-high group were significantly associated with poor prognosis (**Figure 3C**, hazard ratio (HR)=2.404, *p*=0.022). In order to compare the sensitivity and specificity of risk score on the prognosis of patients with breast cancer, time-dependent receiver operating characteristics (ROC) analysis was performed. The area under the ROC curve (AUC) of the ECM group was 0.755 (**Figure 3D**), suggesting the ECM dysregulation prognostic signature for breast cancer was highly reliable. These results together identify ECM dysregulation as a key player in the prognosis of breast cancer.

### C-ECM Dysregulation Is Related With Immunological Activity and ECM Associated Biology Process

**Figure 4:**
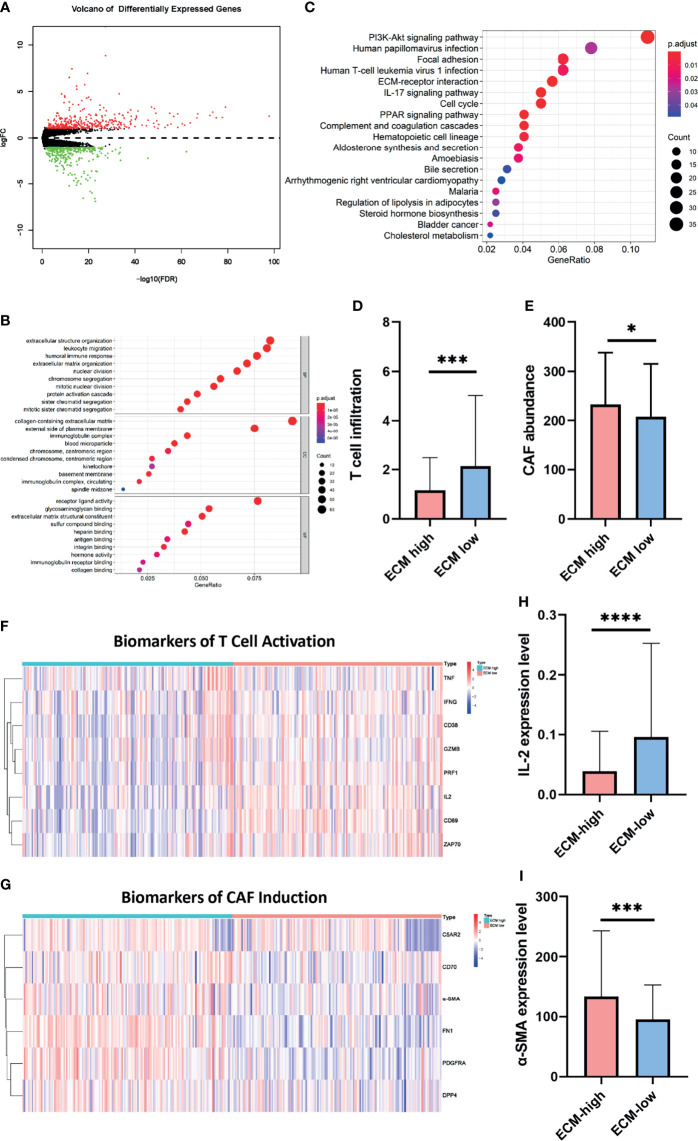
C-ECM dysregulation is associated with T cell and CAF activities. **(A)** Volcano plot of differentially expressed genes across ECM-high and ECM-low groups. **(B)** Results of KEGG analyses across different ECM score groups. **(C)** Results of GO analyses between different ECM score groups into three functional groups, including biological process (BP), cellular component (CC) and molecular function (MF). **(D)** T cell infiltration of ECM-high and ECM-low groups conducted by TIMER. **(E)** Abundance of CAFs of ECM-high and ECM-low groups conducted by TIMER. **(F)** The heatmap of T cell activation biomarkers of ECM-high and ECM-low groups. **(G)** The heatmap of CAF induction biomarkers of ECM-high and ECM-low groups. **(H)** The IL-2 expression level of ECM-high and ECM-low groups. **(I)** The α-SMA expression level of ECM-high and ECM-low groups. *P < 0.05; ***P < 0.001, ****P < 0.0001.

Since our data suggest that the ECM dysregulation was associated with the prognostic of breast cancer, we try to select possible pathways under this dysregulation. To do this, the mRNA expression profiles were compared across the best prognosis (ECM-high) and the worse prognosis (ECM-low) groups. A total of 793 differentially expressed genes including 403 upregulation and 390 down regulation genes were identified (**Figure 4A**). Subsequently enrichment factor of Gene Ontology (GO) and Kyoto Encyclopedia of Genes and Genomes (KEGG) pathway analyses referred to the significance of specific functions. In the GO analysis, DEGs were significantly enriched in ECM associated process such as extracellular structure organization (GO:0043062), collagen-containing extracellular matrix (GO:0062023), extracellular matrix structural constituent (GO:0005201) and so on (**Figure 4B**). Notably, enrichment for inflammatory processes and adaptive immune responses such as humoral immune response (GO:0006959), leukocyte migration (GO:0050900), T cell activation (GO:0042110) immunoglobulin complex (GO:0019814), were also identified (**Figure 4B**). For the KEGG pathway analysis, DEGs were significantly enriched in ECM-receptor interaction, PI3K-Akt signaling, IL-17 signaling, human T-cell leukemia virus 1 infection pathway (**Figure 4C**).

As we identified ECM associated and immune response pathways, we then asked whether those two process were related. Given the previously identified role of CAFs in determining the composition and organization of ECM (19, 20), as well as T cell infiltration and activation in clinical efficacy of immunotherapies (21), we then try to evaluate the association between CAF induction, T cell infiltration and ECM dysregulation. To further support this hypothesis, Micro-environment Cell Populations-counter (MCP-counter) algorithm were conducted by TIMER to examine the abundance of T cell infiltration and CAF populations (16). Importantly, samples in the ECM-high group exhibited lower infiltration of T cells (1.153 vs 2.136, p=0.0001) and higher quantification of CAFs (232.6 vs 207.2, p=0.0343) (**Figures 4D, E**). To further confirm the results, two sets of T cell function and CAF activation biomarker genes were generated. Consistently, T cell function biomarkers were significantly lower in the ECM-high group, while CAF biomarkers were significantly higher (**Figures 4F**–**I**). Collectively, these results indicate that ECM characteristics affect activities of T cells and CAFs.

### ECM Characteristics Are Associated With Breast Tumor Progression and Metastasis in Clinical Samples

**Figure 5:**
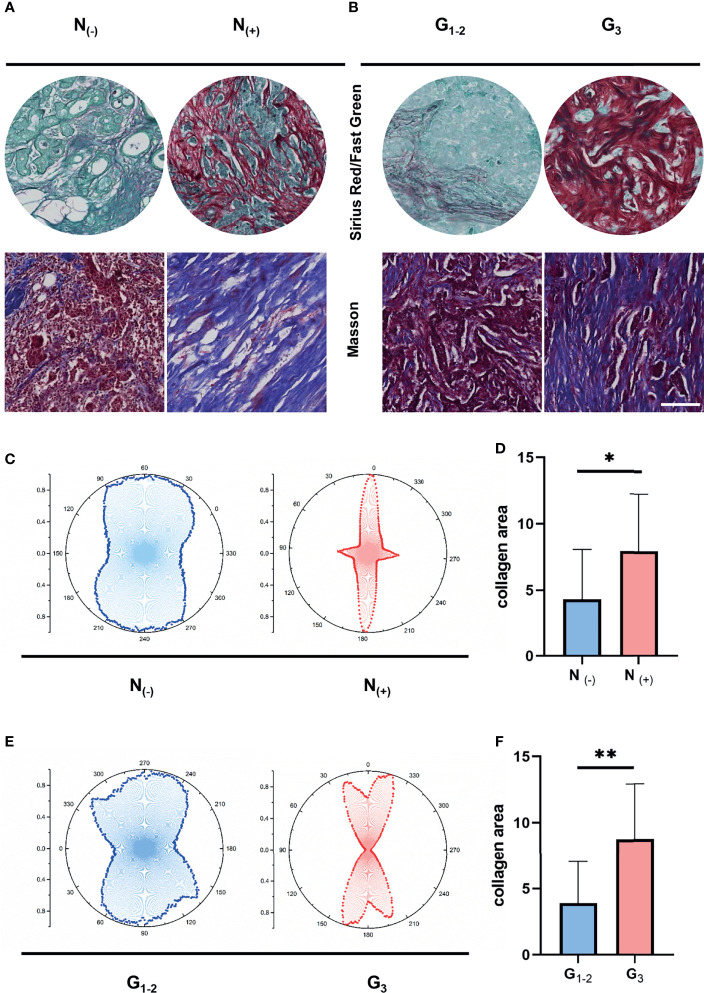
ECM characteristics are associated with breast tumor progression and metastasis in clinical samples. **(A)** Masson’s trichrome and Sirius red/Fast Green staining between different nodal status. **(B)** Masson’s trichrome and Sirius red/Fast Green staining between different pathological stages. **(C)** Orientation distribution of collagen fibrils between different nodal status. **(D)** Area of collagen fibrils between different nodal status. **(E)** Orientation distribution of collagen fibrils between different pathological stages. **(F)** Area of collagen fibrils different pathological stages. (scale bar, 100 μm). *P < 0.05; **P < 0.01.

To understand the corresponding pathological characteristics of ECM dysregulation in human breast cancer samples, we performed Masson staining and Sirius red/Fast green staining on 30 breast cancer samples to evaluate the characteristics of collagen fibrils that constitute the main component of ECM. the clinical information of the real breast cancer patients was list in **Table 1**. We compared the collagen density and organization of breast cancer patients with nodal status negative and positive as well as grade I-II and grade III (**Figures 5A, B**). Interestingly, the results showed increased collagen fibril alignment in the nodal status positive (**Figure 5C**) and grade III groups (**Figure 5E**) contrast to nodal negative or grade I-II breast cancer tissues. In addition, the density of collagen showed higher levels in the nodal status positive (**Figure 5D**) and grade III groups (**Figure 5F**). These results suggest an essential role of ECM characteristics in breast cancer invasion and metastatic dissemination. Give that ECM-high groups showed worse prognosis, taken together, these results strongly support increasing ECM alignment and density promoting invasion and predict worse prognosis in breast cancer.

### Aligned Collagen Matrix Enhances CAF Induction and Inhibits T Cell Activation

**Figure 6:**
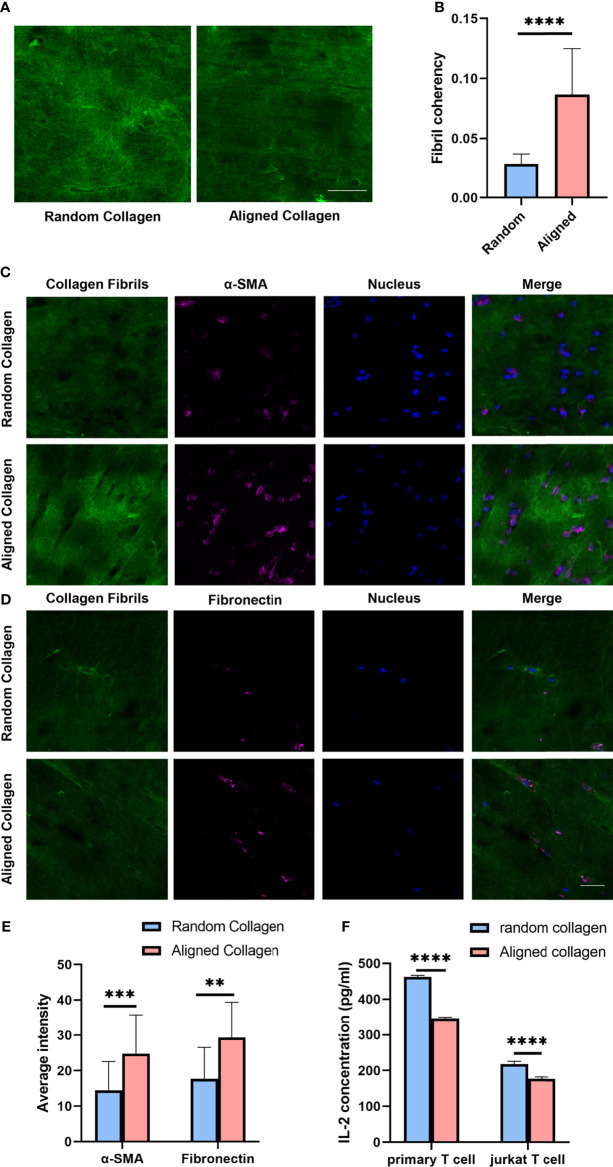
Aligned collagen matrix enhances CAF induction and inhibit T cell activation. **(A)** Random and aligned collagen fibrils were successfully conducted by magnetic stretching. **(B)** Fibril coherency between the random and aligned groups. **(C)** Immunofluorescence staining of α-SMA of MRC-5 cells encapsulated in random and aligned collagen fibrils. **(D)** Immunofluorescence staining of fibronectin of MRC-5 cells encapsulated in random and aligned collagen fibrils. **(E)** Average fluorescence intensity of α-SMA and fibronectin between random and aligned groups. **(F)** IL-2 levels of activated primary T cells and Jurkat T cells encapsulated in random and aligned collagen fibrils. (scale bar, 50 μm). **P < 0.01; ***P < 0.001, ****P < 0.0001.

Considering that the combined results suggests breast cancers with different invasiveness and prognosis showed different ECM characteristics with specific immune status and CAF activity, we tested the hypothesis that ECM characteristics would affect the activity of T cell activation and CAF induction. To evaluate the potential effects of CAF induction and T cell activation by collagen organization, we visualized the α-SMA and fibronectin expression of MRC-5 and NIH/3T-3 cells as well as the secretion of IL-2 of primary T cells and Jurkat T cells in 3D random and aligned collagen matrix supplement with supernatant samples of breast cancer cell MB-231. Visually, through magnetic stretching like **Figure S3A**, collagen fibrils with magnetic stretching remained aligned, whereas the control group remained random organized (**Figures 6A, B**). The α-SMA and fibronectin expression was significantly higher in the aligned collagen matrix (**Figures 6C**–**E** and **Figures S3C, D**). Conversely, the IL-2 levels in activated primary T cells and jurkat T cells exhibited significant decrease in the aligned group (**Figure 6F**). Remarkably, as activated primary T cells and jurkat T cells secreted IL-2 at levels significantly higher than the assay range, we detected IL-2 levels in a 10-fold dilution (**Figure S3B**). These observations indicate that increased ECM alignment significantly promote CAF induction and inhibit T cell activation, thus increase breast cancer invasiveness.

### Increased Collagen Density Promotes CAF Induction and Downregulates T Cell Activation

**Figure 7:**
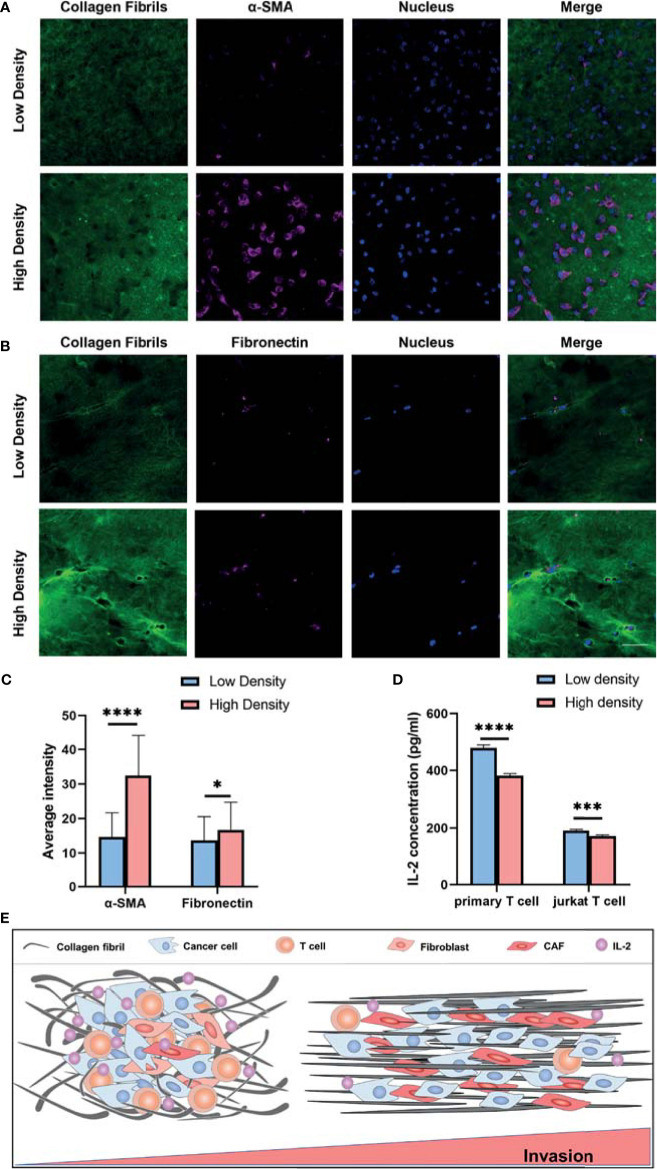
Collagen density modulates the CAF induction and T cell activation. **(A)** Immunofluorescence staining of α-SMA of MRC-5 cells encapsulated in low and high density of collagen fibrils. **(B)** Immunofluorescence staining of fibronectin of MRC-5 cells encapsulated in low and high density of collagen fibrils. **(C)** Average fluorescence intensity of α-SMA and fibronectin between different collagen density groups. **(D)** IL-2 levels of activated primary T cells and Jurkat T cells encapsulated in low and high density collagen fibrils. **(E)** High invasive breast cancer subtypes exhibit increased ECM density and alignment consistency, which promotes tumor invasion due to immunosuppression of T cells as well as CAF activation. ns, not significant; (scale bar, 50 μm). *P < 0.05; ***P < 0.001, ****P < 0.0001.

To further investigate the CAF induction and T cell activation regulated by another ECM characteristic, ECM density, we compared MRC-5 cells, NIH/3T-3 cells, primary T cells and Jurkat T cells cultured in 3D high- and low-density collagen matrix (4mg/ml vs 1mg/ml). We observed that α-SMA and fibronectin expression were significantly upregulated in high-density groups (**Figures 7A**–**C** and **Figures S4A, B**). Consistent with the published study (15), the increased collagen density was accompanied by decreased secretion of IL-2 (**Figure 7D**). Altogether, these results suggest that the T cells acquire a decreased activation in the high density than low density of collagen gels. This is consistent with the TCGA database analysis (**Figure 4G**).

Altogether, our study reveals that breast cancer patients with high ECM scores have increased ECM density and aligned organization, which promotes breast cancer invasion and metastasis *via* immunosuppression of T cells as well as enhancing CAF induction (**Figure 7E**).

## Discussion

ECM characteristics comprise strong prognostic indicators of breast cancer invasion and prognosis (6, 8, 22, 23). However, there is currently no specific study that characterized ECM along the different stages of breast cancer development, thus what kinds of specific ECM characteristics are associated with breast cancer invasion is worth exploring. Additionally, how these invasive subtypes of ECM affect the activities of other important cells in the microenvironment remains poorly understood. In this study, we first identified the associations between ECM dysregulation and prognosis through of breast cancer in the TCGA database, followed by the identification of aggressive ECM subtypes in clinical samples. According to the above ECM characteristics in clinical samples, we established an *in vitro* 3D culture system that is highly similar to the clinical sample by adjusting the concentration and organization of 3D collagen matrix and evaluated T cell activation and CAF induction in the specific ECM microenvironment. Based on the *in vitro* 3D collagen matrix system, we validate the hypothesis that the invasive ECM microenvironment presenting increased collagen density and aligned organization could promote breast cancer invasion *via* inhibiting the activation of T cells and facilitating the induction of CAFs.

Consistent with the previous demonstration (14), we found that high ECM-up scores indicates worse prognosis of breast cancer. We subsequently observed the density and organization of collagen fibrils varied during the progression of breast cancer, with a gradual increase in the deposition and alignment of collagen fibrils. Given that high collagen deposition and alignment contribute to breast cancer invasion through overexpression of EGFR, ERBB2, CD44 and other receptors in the microenvironment, which further induce tumor invasion and metastasis through the transduction of downstream PI3K/Akt, MAPK and other signaling pathways (23–26). Our clinical analysis suggest that the high density and well-aligned collagen fibrils represent a more invasive ECM subtype, and combined with the results from the TCGA database, combined with the results of the TCGA database, we can infer that the higher risk group of patients with a worse prognosis correspond to the invasive ECM subtype. This provides *in vivo* evidence to support our subsequent construction of *in vitro* 3D culture models that more accurately mimic the tissue environment of breast cancer patients.

Another important observation in our study was that induction of CAFs and activation of T cells clearly respond to the deposition and organization of collagen fibrils. More recently, it has been shown that CAFs alter the organization and deposition of the ECM in a variety of ways to remodel the ECM, thereby preventing chemotherapies from reaching tumor cells (27, 28). However, how the reshaped ECM affects the activity of CAFs remains largely unclear. Indeed, we observed increased density and collagen alignment would promote the expression of the CAF biomarker α-SMA and therefore promote CAF induction. Force-dependent collagen fibril alignment can enhance the diffusion of CAF-promoting exosomes to reach the stroma and induce CAFs may be one of the explanations (29). As for collagen density, as the increase in collagen concentration causes an increase in ECM stiffness, which in turn activates the YAP and TAZ transcriptional regulators, thus facilitates the CAF induction (30). GO and KEGG analysis to identify biological process categories that were statistically enriched confirmed that T cell activation was significantly affected by ECM dysregulation. In alignment with these analysis, we observed in *in vitro* 3D culture model that increased collagen density, as well as aligned organization, had a suppressive effect on T cell activation, which may constitute a novel immunosuppressive mechanism within the breast cancer microenvironment. Although this is the first study that directly assess the response of T cell activation to the surrounding ECM density and organization, the potential of ECM characteristics to modulate immune activity is supported by several study, in which ECM stiffness was shown to influence the recruitment of monocytes and their differentiation into protumorigenic macrophage subsets (31). In our study we focused on the ability of the T cell activity, but it is possible that ECM characteristics could further regulate the activities of other cells in the tumor microenvironment, which were worth further exploration as well.

Since increased ECM alignment and density can accelerate breast cancer progression by promoting fibroblast induction and inhibiting T-cell activation according to the combination of clinical samples, database analysis and *in vitro* 3D models, the molecular signal pathway changes under this observation are particularly important. In addition to the ECM-associated and immune-related molecular pathways we mentioned previously, we also identified the ‘PI3K-Akt’ and ‘Focal Adhesion’ signaling pathway *via* KEGG analysis. Given that PI3K-AKT signaling pathway has diverse downstream effects on cellular energy metabolism process (32), added with the previous evidence that ECM stiffening increased the confinement of cell migration within 3D matrix thereby increasing energy consumption (33), we can deduce that increased collagen density and alignment showed the similar effect. During this process, cells can detect and react to changes of ECM through integrin-based adhesion sites *via* mechanotransduction (34). Then the transmission of forces across integrin-based adhesions establishes communication between ECM and cancer cells, which then regulate both rapid responses in cellular energetic metabolism and long-term changes in gene expression (34). However, there are still a lot of open questions that need to be answered to gain a comprehensive understanding of this process.

To our best knowledge, this is the first study that present direct 3D experimental evidence that ECM alignment and density can accelerate breast cancer progression by promoting fibroblast induction and inhibiting T-cell activation, which remind us the potential treatment role of ECM organization and density in breast cancer. Meanwhile, based on the important role of ECM during cancer invasion, an important step of anticancer treatment is the identification of the biological alterations present in tumor microenvironment in order to target these key molecular players. For example, matrix metalloproteinases (MMPs) secreted by CAFs which promote ECM degradation were noted as promising targets against breast cancer (35). Presently, the MMP-14 blocking antibody DX-2400 has been tested in a murine model of breast cancer, where it inhibited primary tumor growth when administered alone and further impaired the growth when combined with radiotherapy (35). Besides, inhibition of FAK represents another opportunity to suppress ECM-induced signaling as downregulation of FAK in breast cancer cells results in decreased tumor growth (19). All these studies accentuate the possibility of using tumor- suppressive ECM components against breast cancer.

## Conclusion

In conclusion, our analyses of the TCGA database and clinical samples revealed a strong correlation between ECM characteristics and breast cancer invasion as well as prognosis. Based on the above results, by encapsulating T cells and fibroblasts in 3D collagen with various densities and organizations, we further demonstrate that ECM characteristics are important regulators of T cell and CAF activity. This immunosuppressive and CAF-induction mechanism could be of central importance for the breast cancer invasion and may constitute a novel therapeutic target to improve breast cancer outcomes. Future studies are needed to explore the in-depth molecular biological mechanisms to identify molecular biomarkers targeting ECM and evaluated the potential efficacy of anti-ECM treatment in breast cancer.

## Data Availability Statement

The datasets presented in this study can be found in online repositories. The names of the repository/repositories and accession number(s) can be found below: https://portal.gdc.cancer.gov, The Cancer Genomic Atlas http://timer.cistrome.org/, TIMER 2.0.

## Ethics Statement

The studies involving human participants were reviewed and approved by Ethics Committee of the First Affiliated Hospital of Xi’an Jiaotong University. The patients/participants provided their written informed consent to participate in this study. The animal study was reviewed and approved by Ethics Committee of the First Affiliated Hospital of Xi’an Jiaotong University.

## Author Contributions

HG and QT contributed to the study design and performed the experiments. LZ, JF, and YZ contributed to data collection. HG, QT, and JY performed statistical analysis and interpretation. HG and JY drafted the manuscript. All authors contributed to the article and approved the submitted version.

## Funding

This study was supported by the National Natural Science Foundation of China (No. 82002794, No.82173277) and Key Research and Development Program of Shaanxi Province (No. 2020GXLHY-018).

## Conflict of Interest

The authors declare that the research was conducted in the absence of any commercial or financial relationships that could be construed as a potential conflict of interest.

## Publisher’s Note

All claims expressed in this article are solely those of the authors and do not necessarily represent those of their affiliated organizations, or those of the publisher, the editors and the reviewers. Any product that may be evaluated in this article, or claim that may be made by its manufacturer, is not guaranteed or endorsed by the publisher.

## Supplementary Material

Supplementary Figure 1 ECM characteristics correlate with breast tumor progression in each molecular subtypes A. The Kaplan-Meier curves for the OS of patients of luminal molecular subtype in TCGA database which were divided into ECM-high, ECM-median and ECM-low group. B. The Kaplan-Meier curves for the DFS of patients of luminal molecular subtype in TCGA database which were divided into ECM-high, ECM-median and ECM-low group. C. The Kaplan-Meier curves for the OS of patients of TNBC molecular subtype in TCGA database which were divided into ECM-high, ECM-median and ECM-low group. D. The Kaplan-Meier curves for the DFS of patients of TNBC molecular subtype in TCGA database which were divided into ECM-high, ECM-median and ECM-low group. E. The Kaplan-Meier curves for the OS of patients of TNBC subtype in TCGA database which were divided into ECM-high and ECM-low group. F. The Kaplan-Meier curves for the DFS of patients of TNBC subtype in TCGA database which were divided into ECM-high and ECM-low group.

Supplementary Figure 2 Forest plots of Univariate Cox regression analysis regarding OS in the TCGA database.

Supplementary Figure 3 Aligned collagen matrix enhances CAF induction of NIH/3T-3 cells A. Random and aligned collagen fibrils models conducted by magnetic stretching. B. IL-2 levels of primary T cells and Jurkat T cells in un-activated and activated groups. C. Immunofluorescence staining of α-SMA of NIH/3T-3 cells encapsulated in random and aligned collagen fibrils. D. Immunofluorescence staining of fibronectin of NIH/3T-3 cells encapsulated in random and aligned collagen fibrils. (scale bar, 50 μm). **P* < 0.05; ***P* < 0.01; ****P* < 0.001, *****P* < 0.0001.

Supplementary Figure 4 Collagen density modulates CAF induction of NIH/3T-3 cells. A. Immunofluorescence staining of α-SMA of NIH/3T-3 cells encapsulated in low and high density of collagen fibrils. B. Immunofluorescence staining of fibronectin of NIH/3T-3 cells encapsulated in low and high density of collagen fibrils. (scale bar, 50 μm).
