# Supplementary figures and images for: 3D Extracellular Matrix Regulates the Activity of T Cells and Cancer Associated Fibroblasts in Breast Cancer
Source: Front Oncol. 2021 Dec 9;11:764204. doi: 10.3389/fonc.2021.764204 (PMC8699235; doi:10.3389/fonc.2021.764204)

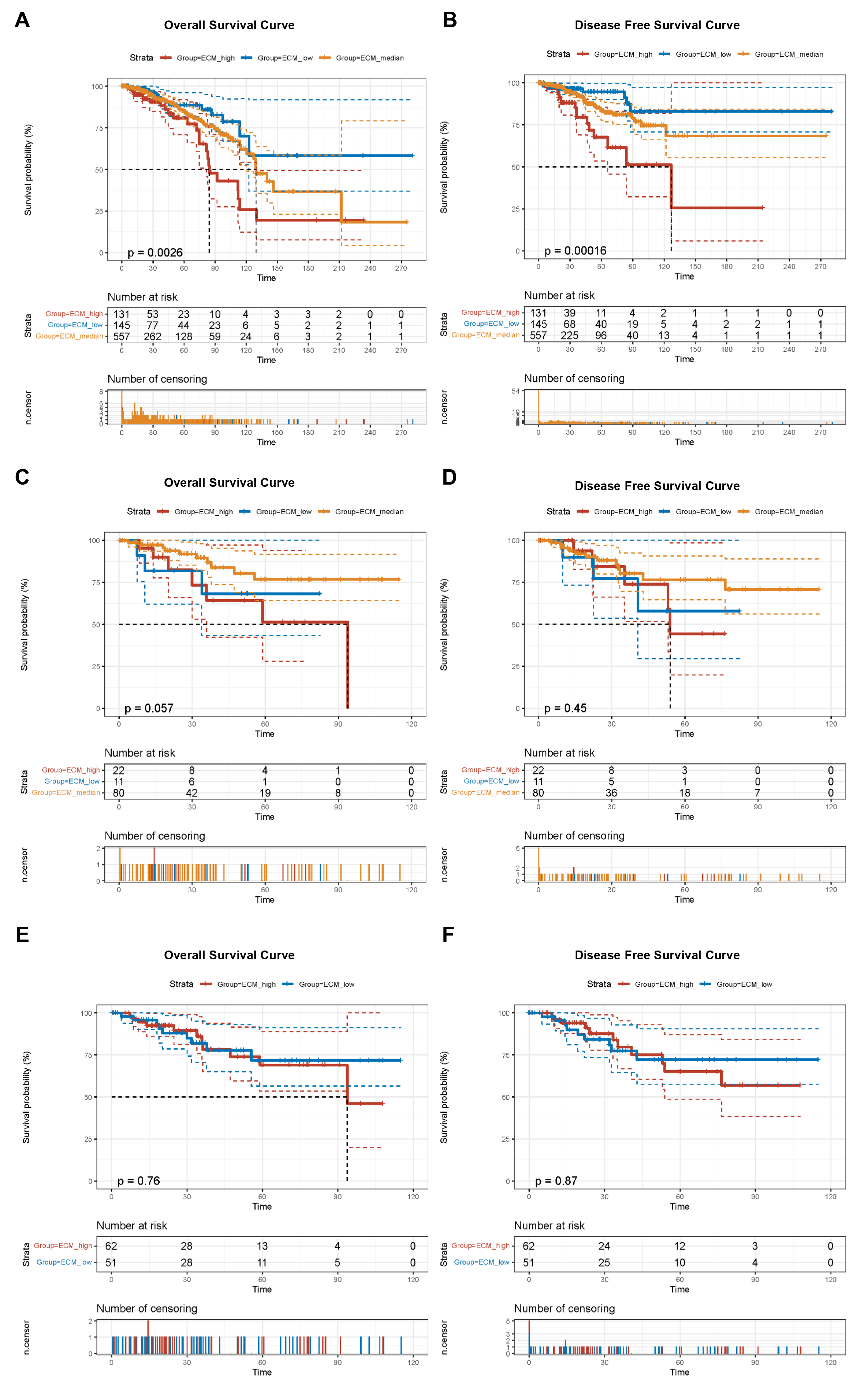

Supplement: Supplementary Figure 1 — ECM characteristics correlate with breast tumor progression in each molecular subtypes A. The Kaplan-Meier curves for the OS of patients of luminal molecular subtype in TCGA database which were divided into ECM-high, ECM-median and ECM-low group. B. The Kaplan-Meier curves for the DFS of patients of luminal molecular subtype in TCGA database which were divided into ECM-high, ECM-median and ECM-low group. C. The Kaplan-Meier curves for the OS of patients of TNBC molecular subtype in TCGA database which were divided into ECM-high, ECM-median and ECM-low group. D. The Kaplan-Meier curves for the DFS of patients of TNBC molecular subtype in TCGA database which were divided into ECM-high, ECM-median and ECM-low group. E. The Kaplan-Meier curves for the OS of patients of TNBC subtype in TCGA database which were divided into ECM-high and ECM-low group. F. The Kaplan-Meier curves for the DFS of patients of TNBC subtype in TCGA database which were divided into ECM-high and ECM-low group. [file Image_1.tif]

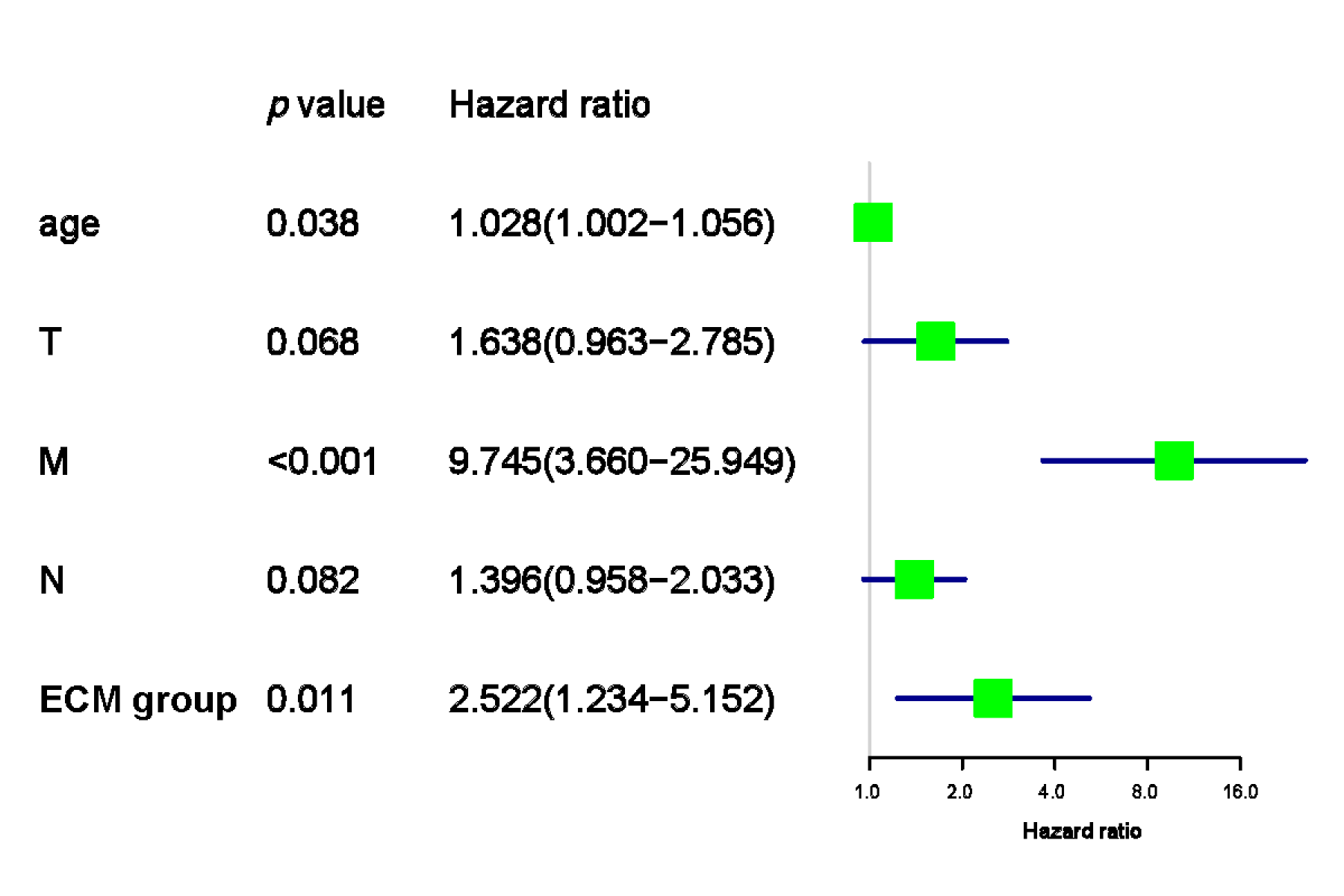

Supplement: Supplementary Figure 2 — Forest plots of Univariate Cox regression analysis regarding OS in the TCGA database. [file Image_2.tif]

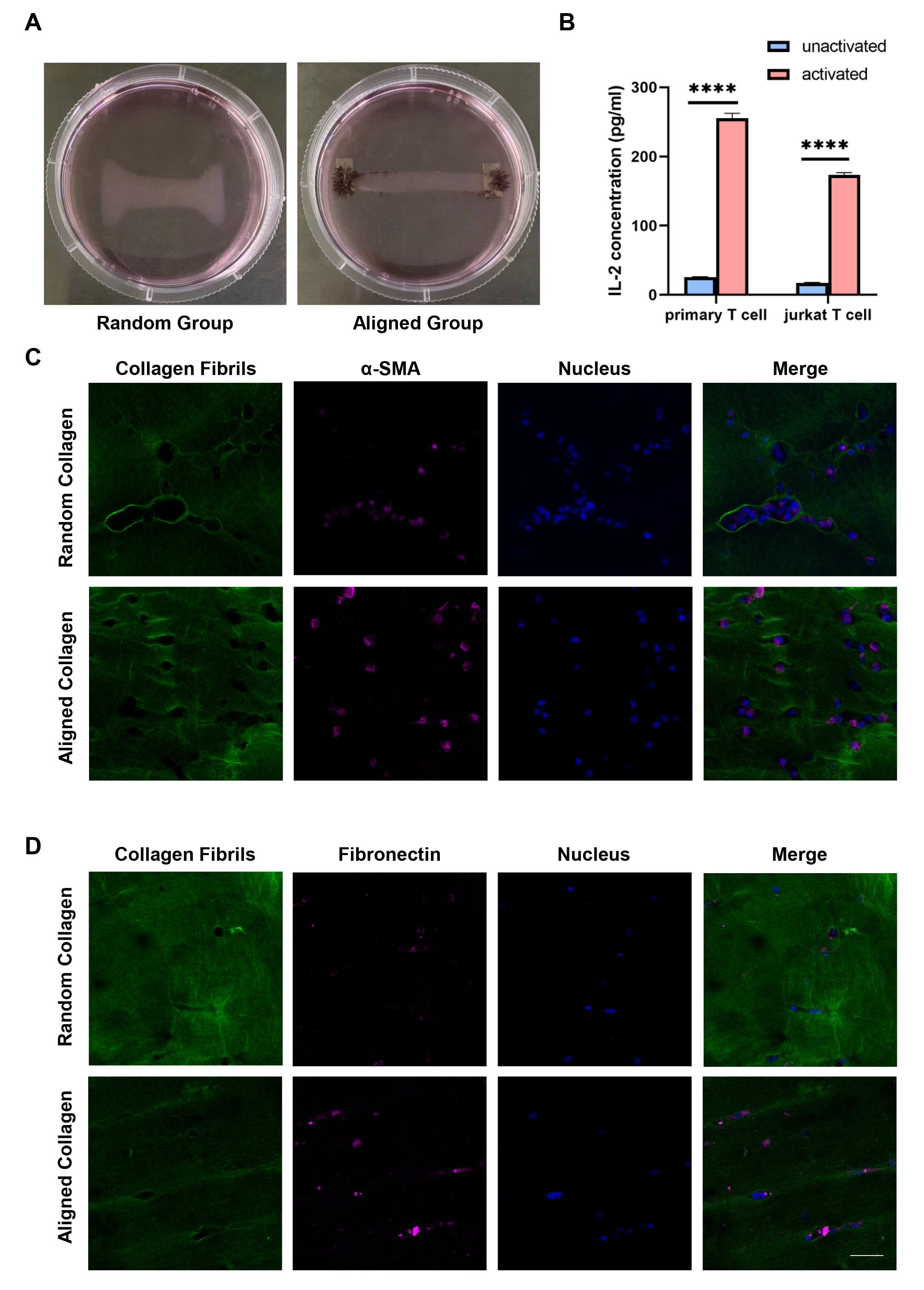

Supplement: Supplementary Figure 3 — Aligned collagen matrix enhances CAF induction of NIH/3T-3 cells A. Random and aligned collagen fibrils models conducted by magnetic stretching. B. IL-2 levels of primary T cells and Jurkat T cells in un-activated and activated groups. C. Immunofluorescence staining of α-SMA of NIH/3T-3 cells encapsulated in random and aligned collagen fibrils. D. Immunofluorescence staining of fibronectin of NIH/3T-3 cells encapsulated in random and aligned collagen fibrils. (scale bar, 50 μm). *P < 0.05; **P < 0.01; ***P < 0.001, ****P < 0.0001. [file Image_3.tif]

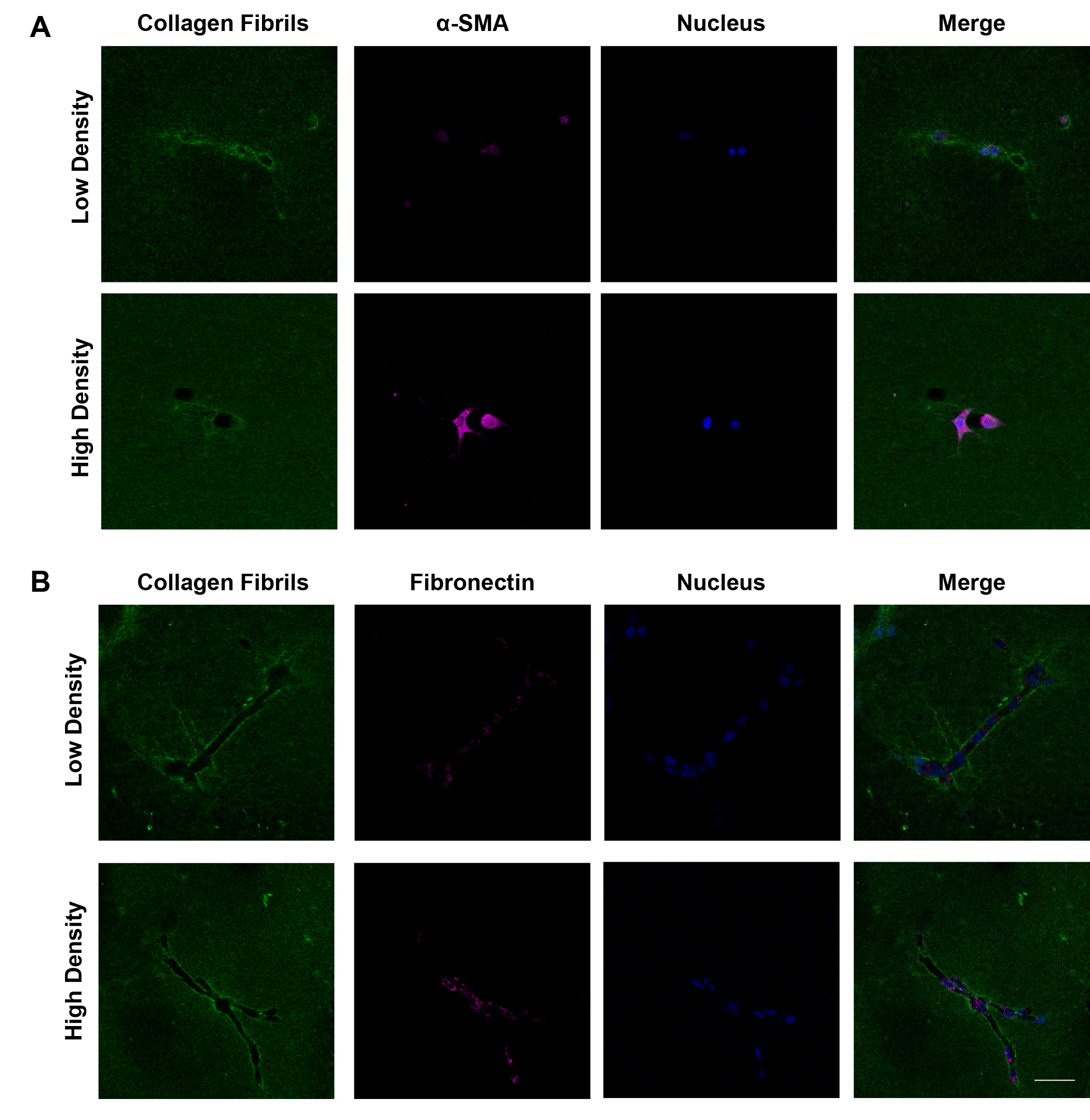

Supplement: Supplementary Figure 4 — Collagen density modulates CAF induction of NIH/3T-3 cells. A. Immunofluorescence staining of α-SMA of NIH/3T-3 cells encapsulated in low and high density of collagen fibrils. B. Immunofluorescence staining of fibronectin of NIH/3T-3 cells encapsulated in low and high density of collagen fibrils. (scale bar, 50 μm). [file Image_4.tif]
